# The colon cancer screening behaviours survey for South Asians: a pilot study of feasibility and psychometric evaluation

**DOI:** 10.1186/s41687-019-0160-z

**Published:** 2020-02-13

**Authors:** Joanne Crawford, Frederick Morfaw, Farah Ahmad, Lehana Thabane, Angela Frisina

**Affiliations:** 1 Faculty of Applied Health Sciences, Department of Nursing, Brock University, St. Catharines, 1812 Sir Isaac Brock Way, St. Catharines, ON L2S 3A1 Canada; 2 Department of Health Research Methods, Evidence, and Impact, McMaster University, Hamilton, ON Canada; 3 School of Health Policy & Management, York University, Toronto, ON Canada; 4 School of Nursing, McMaster University, Hamilton, ON Canada

**Keywords:** Colorectal cancer, Early detection of cancer, South Asian, Pilot testing, Survey instrument, Psychometric properties, Canada

## Abstract

**Purpose:**

The purpose of the study was to pilot test the English and Urdu version of the Colon Cancer Screening Behaviours Survey among South Asians in Canada. The first objective was to evaluate feasibility of administration, data collection using computer assisted personal interviewing software on a tablet, and response burden. The second objective was to examine the prevalence of colorectal cancer screening among South Asians and evaluate the psychometric properties of sub-scales in the survey.

**Methods:**

Purposive, network and snowball sampling were used to recruit participants for this cross-sectional study. Interviewer-led administration of the Colon Cancer Screening Behaviours Survey was conducted across two cities in Ontario, Canada. Qualitative data analysis assessed feasibility; and sub-scales were evaluated through principal component analysis, item-scale correlations, and construct validity using multiple linear and logistic regression.

**Results:**

A total of 328 South Asians participated, 47% Urdu speaking, and 53% English speaking. There was a 23% refusal rate to participate. Feasibility identified: (1) successful recruitment despite reasons for refusal; (2) problematic items and response categories; and (3) computer/tablet limitations. Principal component analysis identified 14 components that explained 68.7% of total variance; 34 items were retained after factor analysis. Internal consistency of 4 scales ranged from 0.79–0.91. There were significant differences in *perceived barriers* scale scores (− 12.21; 95% CI, − 17.13 to - 7.28; *p* <  0.0001) between those who participated and those who did not participate in screening. No association was found with years of residence and uptake of screening after adjustment (OR 0.91 (0.46–1.79), *p* = 0.783).

**Conclusions:**

Recruitment and data collection methods are feasible among South Asians if functionality of the tablet selected is improved. The Colon Cancer Screening Behaviours Survey was finalized and retained items in sub-scales demonstrated good psychometric properties to assess behaviours for colon cancer screening among South Asians in Canada. The interviewer-led survey may be used by public health, cancer care or other health practitioners to describe or predict colorectal cancer screening behaviours among South Asians in similar settings or adapted and tested in other contexts.

## Introduction

Worldwide, colorectal cancer (CRC) is one of the top three cancers diagnosed among men and women [1]. CRC incidence is lower in countries in transition, such as South Asian countries of India, Sri Lanka and Pakistan; however, this is changing. In Sri Lanka, CRC is the fourth most common cancer diagnosed in men [2, 3]. Increasing CRC trends in South Asian countries may also influence the risk of CRC among South Asians (SAs) who settle in other countries including those in the west, especially when evidence shows that the “healthy immigrant effect” tends to diminish with long term residence [4]. Studies in Canada, for example, document that the patterns of cancer incidence among SAs approach native-born Canadian populations [5]. Hislop et al. [5] report increased rates of CRC among SAs in Canada compared to their counterparts from India, although rates remained lower than native-born Canadians. Likewise, CRC incidence is lower among immigrants from South Asia when compared to populations originating from East Asia, the Pacific, Europe, and Central Asia [6]. It is estimated that the risk for CRC increases by 5–7% every 5 years with time of settlement [6]. This gradual increased risk resembles the current patterns of incidence in the general population given that CRC among SAs in Canada accounted for one of the top three cancers diagnosed [5]. Comparable trends of CRC incidence are noted among SAs in the United States (US) and the United Kingdom (UK) [7–10].

Organized CRC screening programs detect and remove polyps or identify early cancer for potential cure, if all average risk individuals participate regularly in biennial screening [11]. Average risk refers to individuals 50–74 years of age with no: personal or family history of CRC or precancerous polyps; hereditary syndromes; inflammatory bowel diseases; or current bowel signs or symptoms [12, 13]. Despite implementation of CRC screening programs in Canada, participation rates continue to fall short of the target of 60% uptake [14, 15]. Fecal occult blood test (FOBT) uptake was 30.1% across provinces with a slight increase to 36.9% for provinces with organized CRC screening programs [15]. Low rates of breast and cervical cancer screening among SAs settled in Canada have been reported in the literature [16–19]; however, CRC screening rates among SAs in Canada is limited. CRC screening rates are lower among recent immigrants compared to long term residents [20] and in communities highly populated with SAs [21]. In Canada, administrative databases do not make distinctions according to ethno-cultural background, and limits understanding of priority groups’ screening status [22].

Our understanding of CRC screening in Canada is based on studies that categorize SAs within broader categories of recent and long-time residents [20, 21]. Yet, it does not tell us about the differences by ethnocultural background or reasons for or against screening. Evidence points to lower likelihood of screening among SAs [23]. Most SAs were under-screened irrespective of having a physician of the same socio-cultural background, and those with lower socio-economic status had lower likelihood of screening [23]. Despite making substantial scholarly contributions, the study by Lofters et al. [23] was limited in identifying the reasons for patients being screened or not, an important gap that still needs to be addressed. Furthermore, there is limited research examining beliefs and attitudes related to CRC screening among SAs in Canada.

With the aim of advancing scholarly work in this area, research was undertaken to explore beliefs, attitudes, and reasons for CRC screening among SAs in Canada [24, 25]. This body of research served to inform development of the Colon Cancer Screening Behaviours Survey (CCSBS) [26]. The survey has six scales, *perceived susceptibility*, *perceived severity*, *perceived benefits, perceived barriers, self-efficacy,* and *subjective norm*. The purpose of the survey is to describe or predict CRC screening that influences uptake among SAs. The CCSBS was cross-culturally adapted into the Urdu language and its cognitive testing demonstrated good content validity [27]. The primary objective of the current study is to evaluate feasibility of survey administration, data collection using computer assisted personal interviewing (CAPI) software, and response burden; and the secondary objectives are to examine psychometric properties of sub-scales along with prevalence of CRC screening among SAs in Ontario, Canada.

## Methods

A cross sectional design was used to administer the CCSBS to average risk SAs in two mid-sized metropolitan cities in Ontario, Canada. Site A has a population of 540,000 and is home to the 3rd highest foreign-born proportion in Canada. Based on 2016 census, SAs were the largest visible minority group in the city [28]. Site B has a population of almost 600,000 and growing in visible minorities, with over 50% of the population born outside of Canada [29]. SAs are also the largest population settling in this city.

### Sample and setting

Random sampling utilized in cross sectional design was not possible given that identification of SA by surname lists in Ontario, Canada would not include all subgroups [30]. SAs were recruited using purposive, snowball, and network sampling. A priori sample size was determined to assess psychometric properties of scales by examining the ratio of participants to parameter to be estimated for a 60-item factor analysis [31], resulting in a sample size of 300. Given that prior cognitive testing was performed [27], we believed items were conceptually consistent across Urdu and English language surveys; thus, this sampling frame was satisfactory to meet study objectives.

Inclusion criteria were: (1) SAs from the Indian sub-continent (e.g. India, Sri Lanka, Pakistan, or other) and *diaspora* such as SAs from South Africa [32]; Urdu or English language ability; (3) 50–74 years of age; (4) permanent resident; and (5) average risk for CRC [12, 13]. Those who were temporary residents or had active symptoms for CRC were excluded.

**Fig. 1:**
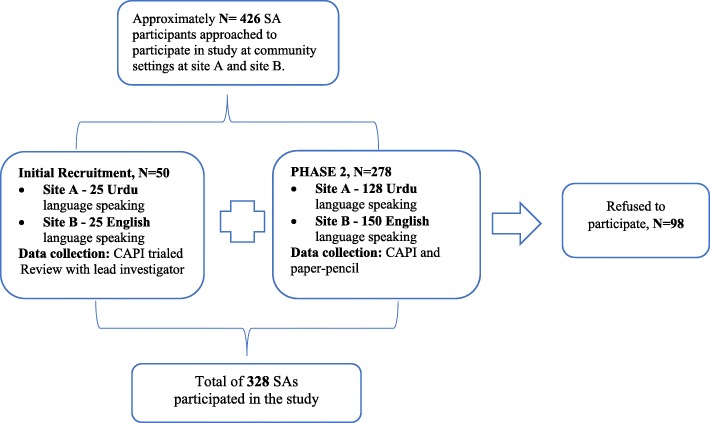
Flow Chart of Participants

Participants were recruited from May to December 2017: Site A, Urdu surveys; and Site B, English surveys (Fig. 1). Recruitment sites included: Temples and places of prayer, recreation centers, local neighbourhood parks, recreation and community centers, cultural festivals and events, and other social gathering places.

### Ethics

Research Ethics approval was received from three university boards by December 2016: (1) Brock University, number 16–033; (2) McMaster University, number 2445; and (3) York University, number 2016–312. Informed consent was provided, and verbal or written consent was obtained from all participants included in the study.

### Survey instrument

The CCSBS, an 84-item survey is divided into four sections: (1) and (2) assesses knowledge, uptake, and intention for the home stool test and colonoscopy; (3) measures beliefs and attitudes about CRC and screening with six sub-scales; and (4) captures socio-demographic characteristics (See Table 1). Summative scales incorporate varying number of items that add up to a total summary score providing data on the strength and direction of the participant’s attitudinal response for a specific construct. Survey administration and data collection employed CAPI software, Survey System 12.0, password protected on two computer tablets. An alphanumeric code was used as the identifier for each participant for CAPI data entry and on paper surveys.

**Table 1:** Colon Cancer Screening Behaviours Survey

Survey Sections	Survey Items = Total of 84 items
Section 1	Items on the home stool test assessing knowledge, uptake, and overall intention to have screening in the future. (items 1–6)
Section 2	Items on the colonoscopy procedure assessing knowledge and uptake, and intention to have a procedure. (7–9)
Section 3	Six multi-item **scales** (*perceived susceptibility, perceived severity, perceived benefits, perceived barriers, perceived self-efficacy*, and *subjective norm*) relevant to colon cancer screening. (items 10–69) • *Perceived susceptibility* included 6 items that assessed self-perceived risk of a diagnosis of CRC. • *Perceived severity* was assessed with 9 items and related to serious nature of CRC, its impact and evaluation if not treated. • *Perceived benefits* (home stool test and colonoscopy) was divided up into 6 items for home stool test and 6 items for colonoscopy examining the benefit to having either test. • *Perceived barriers* (home stool test and colonoscopy) included 11 items for the home stool test and 10 items for colonoscopy and represented the factors that impeded decisions to have CRC screening. • *Perceived self-efficacy* was measured with 7 items and assessed an individual’s confidence in performing the home stool test or preparing for colonoscopy. • *Subjective norm* was measured using 5 items and examined the social influences and the individuals desire to comply with having CRC screening. **Response Categories:** 7-point Likert Scale for response categories: 1 – strongly disagree, 2 – moderately disagree, 3 – mildly disagree, 4 – neither disagree or agree, 5 – mildly agree, 6 – moderately agree, 7 – strongly agree
Section 4	Population socio-demographic items (70–84)
Crawford et al. [26, 27]	

### Procedures

The CCSBS was developed for interviewer-led administration by SA Research Assistants (RAs) to increase access for those with literacy barriers, assist with difficult questions, minimizes non-response, and improve data collection [33]. RAs required training on colon cancer and screening, local and regional resources, how to respond to participants, and when to contact the lead investigator. This also included training on CAPI software, ethical conduct, and a mock interview.

Once eligibility was established, the RAs provided information and discussed confidentiality. Interested participants were provided with informed consent. Some participants chose to discuss the study with family and were given the RA contact information. This allowed us to respect the collectivist beliefs of SAs and their decision-making processes. To monitor interview consistency and data collection, ongoing communication and interaction with RAs occurred at completion of 50, 100, and 200 surveys with the lead investigator, and as needed. All collected data was transferred onto an Excel spreadsheet for verification and data cleaning. The cleaned dataset was then transferred into the Statistical Package for the Social Sciences (SPSS) version 21.0 for analysis.

### Data analysis

Descriptive statistics were used to summarize socio-demographic factors, and CRC screening prevalence and intention to screen using means (standard deviations) and frequency (percentages).

### Reliability

Principal component analysis assessed internal scale structure of six scales. Orthogonal rotation of extracted factors was completed using normal varimax procedure. Eigenvalues of 1.0 or greater were retained based on Kaiser criterion [34]. The scree test was used to confirm components to retain. Principal component analysis was performed to assess factor loadings for each of the items on the principal components identified. Items loading 0.30 or higher on components were retained [35]. The final factor solution was used to conduct reliability and validity analyses.

Internal consistency reliability for each sub-scale was estimated using Cronbach’s alpha. An alpha less than 0.20 represents items within the scale that are unrelated and measure other concepts; whereas, alphas over 0.8 represent items within the scale that may be redundant because of higher correlation [35]. The parameters used in assessing Cronbach alpha was to retain items that were 0.7 or above in any of the sub-scales. Prior to final removal of each individual item, Cronbach’s alpha of items within each sub-scale were examined along with a conceptual review for final determination to remove an item from the specific sub-scale. Item-to-total correlation (ITC) assessed items and correlation to other items in the scale; ITC estimates of 0.2 or greater were retained [35, 36].

Test-retest reliability was impractical due to the community recruitment process and potential challenges with completing the second survey administration in this population [37].

### Construct validity

We used multiple linear regression to assess construct validity for variables reported on a continuous scale, to examine *perceived barriers* scale scores in those who had and did not have CRC screening, while adjusting for age, years lived in Canada, gender, country of birth, ethnic background, marital status, level of education, language, employment status, family household, household income, and having a family doctor. We report the beta coefficients and associated 95% confidence intervals. To assess construct validity for CRC screening adherence among recent and long-term residents, we used logistic regression as this variable was reported on a binary scale. We adjusted for the same covariates as above. For this variable specifically, we report the odds ratio and 95% confidence intervals.

While factor analysis is one form of construct validity testing; known group comparison may also serve to examine construct validity [35]. Studies have demonstrated that low self-*perceived barriers* are associated with increased uptake of CRC screening [38, 39]. Therefore, our hypothesis was that participants who report CRC screening adherence (participation in CRC screening using the home stool test or colonoscopy) would have lower *perceived barriers* scale scores compared to those who do not report CRC screening adherence. Length of residence in Canada has also been tied to CRC screening uptake. A second hypothesis was that participants in Canada for ≥10 years would have greater CRC screening uptake than those who resided in Canada ≤10 years [20].

## Results

### Description of the sample

A total of 328 SAs participated; 47% Urdu (Site A), and 53.4% English (Site B). Refusal rate of participants approached to participate was approximately 23% (Fig. 1). Mean age was 60.7 years. Site A had 13% more females, whereas Site B had 15% more males (See Table 2). India was the main origin country (Site A, 67%; Site B, 92%); Site A had greater diversity in country of origin than Site B. Most participants lived with family members, and more than half did not know or preferred not to disclose household income.

**Table 2:** Socio-demographic characteristics

Variable (*N* = 328)	Urdu (Site A) (Hamilton) *n* = 153 (46.6%)	English (Site B) (Brampton) *n* = 175 (53.4%)	Overall
Age (Years): Mean (SD)	60.7 (7.7)	60.6 (7.2)	60.7 (7.4)
Years lived in Canada: Mean (SD)	23.7 (13.8)	19.3 (10.4)	21.4 (12.2)
Gender: *n* (%)			
Male	67 (43.8)	101 (57.7)	168 (51.2)
Female	86 (56.2)	74 (42.3)	160 (48.8)
Country of Birth: *n* (%)			
India	67 (43.8)	161 (92.0)	(228) 69.5
Pakistan	55 (35.9)	3 (1.7)	(58) 17.7
Bangladesh	2 (1.3)	0 (0.0)	(2) 0.6
Other	29 (19.0)	11 (6.3)	(40) 12.2
Ethnic background: *n* (%)			
Punjabi	11 (7.2)	8 (4.6)	19 (5.8)
Sri Lankan	0 (0.0)	3 (1.7)	3 (0.9)
Gujarati	50 (32.7)	1 (0.6)	51 (15.5)
Pakistani	30 (19.6)	1 (0.6)	31 (9.5)
Bengali	2 (1.3)	0 (0.0)	2 (0.6)
Other [Sikh, Hindu, Muslim, Kutchi, Persian, Rathi, Trinidadian, West Indian]	9 (6.1)	8 (4.6)	11 (5.1)
Punjabi and Sikh	22 (14.4)	146 (83.4)	168 (51.2)
Marital status: *n* (%)			
Married	132 (86.3)	159 (90.9)	291 (88.7)
Living as married	1 (0.7)	3 (1.7)	4 (1.2)
Divorced	4 (2.6)	2 (1.1)	6 (1.8)
Separated	1 (0.7)	0 (0.0)	1 (0.3)
Widow	12 (7.8)	11 (6.3)	23 (7.0)
Never married	2 (1.7)	0 (0.0)	2 (0.6)
Have a partner but do not live with them	1 (0.7)	0 (0.0)	1 (0.3)
Highest level of education: *n* (%)			
Less than high school	22 (14.4)	18 (10.3)	40 (12.2)
Completed high school	28 (18.3)	48 (27.4)	76 (23.2)
Completed some college/University	49 (32.0)	41 (23.4)	90 (27.4)
Completed trade, certificate, or diploma	16 (10.5)	9 (5.1)	25 (7.6)
University certificate/diploma	7 (4.6)	17 (9.7)	24 (7.3)
University degree (Bachelor’s)	25 (16.3)	19 (10.9)	44 (13.4)
Post graduate degree	6 (3.9)	23 (13.1)	29 (8.8)
Employment status: *n* (%)			
Employed outside the home full-time (> 30 h /week)	43 (28.1)	78 (44.6)	121 (36.9)
Employed outside the home part-time (up to 30 h /week)	15 (9.8)	26 (14.9)	41 (12.5)
Looking after a home/family	8 (5.2)	3 (1.7)	11 (3.4)
Unemployed	16 (10.5)	3 (1.7)	19 (5.8)
Not working but seeking work	2 (1.3)	0 (0.0)	2 (0.6)
Retired	48 (31.4)	63 (36.0)	111 (33.8)
On disability or government program	1 (0.7)	1 (0.6)	2 (0.6)
Other	20 (13.1)	1 (0.6)	21 (6.4)
Individuals who make up your family: *n* (%)			
Lives with partner and children	80 (52.3)	63 (36.0)	143 (43.6)
Lives with children only	21 (13.7)	16 (9.1)	37 (11.3)
Lives with children and in laws	3 (2.0)	35 (20.0)	38 (11.6)
Lives with partner only	37 (24.2)	33 (18.9)	70 (21.3)
Lives with partner, and in laws	4 (2.6)	13 (7.4)	17 (5.2)
Lives with partner, children and parents	3 (2.0)	13 (7.4)	16 (4.9)
Lives alone	5 (3.3)	2 (1.1)	7 (2.1)
Annual household income before tax (CAD): *n* (%)			
< 19,999	14 (9.2)	23 (13.1)	37 (11.3)
20,000–29,999	16 (10.5)	0 (0.0)	16 (4.9)
30,000–39,999	11 (7.2)	3 (1.7)	14 (4.3)
40,000-49,999	12 (7.8)	9 (5.1)	21 (6.4)
50,000-59,999	4 (2.6)	15 (8.6)	19 (5.8)
60,000-79,999	8 (5.2)	10 (5.7)	18 (5.5)
80,000- 99,999	3 (2.0)	8 (4.6)	11 (3.4)
More than 100,000	2 (1.3)	6 (3.4)	8 (2.4)
Don’t know or prefer not to answer	83 (54.2)	101 (57.7)	184 (56.1)
Yes	145 (94.8)	175 (100.0)	320 (97.6)
No	8 (5.2)	0 (0.0)	8 (2.4)
Gender of family doctor (*n* = 320)_a_			
Female	74 (48.4)	42 (24.0)	116 (36.3)
Male	71 (46.4)	133 (76.0)	204 (63.7)
Family doctor of similar culture as client (n = 320) _a_			
Yes	69 (45.1)	152 (86.9)	221 (69.1)
No	76 (49.7)	23 (13.1)	99 (30.9)
Preference of gender of health care provider			
Male	23 (15.0)	22 (12.6)	45 (13.7)
Female	53 (34.6)	34 (19.4)	87 (26.5)
No preference	77 (50.3)	119 (68.0)	196(59.8)
Other cancer screening test done in females only: *n* (%)			
Mammography only	2 (13.1)	4 (2.3)	24 (7.3)
Pap smear only	1 (0.7)	4 (2.3)	5 (1.5)
None	12 (7.8)	3 (1.7)	15 (4.6)
Not applicable	67 (43.8)	101 (57.7)	168 (51.2)
Both Mammography and Pap smear	53 (34.6)	63 (36.0)	116 (35.4)

*SD* Standard deviation. a: only for clients with family doctors (*n* = 320)

Self-report uptake of home stool test was lower in Site A, 51.6% compared to Site B, 66.3% For self-report colonoscopy screening, uptake was higher at Site A, 35.9% compared to Site B, 18.3%. When asked about intention to have future CRC screening, 68.3% indicated that they planned to have a home stool test in the future (Site A, 46.4% versus Site B, 87.4%). See Table 3.

**Table 3:** Colorectal cancer screening uptake and intention

History of a colorectal cancer screening test	Percentage in Brampton (95% CI)	Percentage in Hamilton (95% CI)	Overall percentage (95% CI)
CRC screening using a “home” test?	66.3 (58.7, 73.1)	51.6 (43.5, 59.7)	59.5 (53.9, 64.8)
CRC screening using colonoscopy?	18.3 (13.0, 24.9)	35.9 (28.5, 44.1)	26.5 (21.9, 31.7)
CRC screening using either “home” test or colonoscopy	68.6 (61.0, 75.3)	60.8 (52.5, 68.5)	64.9 (59.5, 70.1)

### Feasibility evaluation

After piloting the first 25 Urdu and 25 English surveys, RAs identified problems with recruitment and data collection. For recruitment, participants did not want to disclose personal information, and sometimes did not trust the RA (Site B). To ameliorate this, we reviewed methods of conveying the importance of confidentiality and collected postal codes instead of addresses. Building trust was more involved, and thus, the RA (Site B) was encouraged to spend more time engaging with community before actively recruiting. Components of evaluation and outcomes are presented (See Table 4).

**Table 4:** Feasibility Outcomes

Evaluation	Outcome	Criteria for success	Results	Lessons Learned
Initial 50 surveys	Less refusal to participate in study when we allowed postal codes to be used. RAs community engagement actions successful in further recruitment efforts.	Recruitment of 25 participants at Site A, and Site B completed in 1 month.	50 participants recruited in 2 months.	Use postal codes in survey with SAs. Community engagement is essential in SA communities. Spending time in setting is required for 1 month before recruitment begins to connect with leaders.
Recruitment	Reinforced confidentiality and anonymity resulted in participants more willing to participate in study.	125 participants for site A and 125 for site B to be recruited in 4 months. Sample size of 300 participants to be recruited.	278 participants recruited in 6 months from both site A and site B. Total sample recruited was 328.	Community-based recruitment using snowball and network sampling takes more time within SA communities. Recruitment target times should be more realistic to the population.
Data collection	RA reinforced rationale for items that were perceived to be similarly worded. Some participants believed the survey took longer to complete than it did, although, it was not a deterrent from participating. Some participants reported too many response categories; and, most felt the midpoint response was acceptable.	Items within each subscale acceptable. Participant survey to be completed within 30 min.	All items in subscales completed during data collection. The average time to complete survey was 15 to 30 min.	Survey administrators need to explain a priori that items may seem to be similar, but they are tapping different aspects of a construct. Reinforce to participants survey length and rationale prior to administration. “I neither agree or disagree” response category is a good midpoint response to use in a Likert Scale with SAs.
CAPI software & resources	The computer/tablet functionality and handling issues were problematic during initial recruitment.	Data collected on computer/tablet is seamless. Visual aids and honorariums acceptable.	RAs used paper-pencil administration alongside CAPI after initial round. Visual aids well received, and honorariums acceptable.	To allow more seamless administration, a better-quality tablet (i.e. enhanced font) would be required for CAPI software use. Use visual aids and provide honorariums for SA participants.

#### Evaluation of recruitment

Community leaders were instrumental to access participants; however, there were refusals. Not knowing the RA or not being introduced to the community were main reasons for refusal to participate. Refusal was somewhat reduced with our decision to accept postal codes and reinforcing confidentiality; yet, some participants continued to refuse because they did not feel comfortable sharing information. Participants who believed they lacked information to complete the survey, asked to consult children or family before participation. Some refusals were related to not being sure how information collected would be used. Other participants not up to date with CRC screening were concerned that we would share this information with their physician, and in turn, they would get into “trouble”. Finally, refusal occurred when participants were told the survey would take 30 min.

#### Evaluation of data collection

Despite the robustness of survey development, there were problematic items or response categories. We report on issues identified below highlighting variations by site (language). Some participants believed items in a measure were similarly worded; for example, item 11 and 12 in the *perceived susceptibility* scale, “I feel I will get colon cancer in the future” and “There is a good possibility I will get colon cancer in the next 10 years”. For Site A participants, the future and the next 10 years were perceived to be the same. The term “distasteful” in the *self-efficacy* scale was problematic for approximately 52% of all participants (Sites A & B); “I am confident that I would not find the home stool test distasteful.” Participants interpreted the “taste” part of the word “distasteful” literally; this item was not concerning because the *self-efficacy* scale was removed altogether. Site B found item 11, “I feel I will get colon cancer in the future” problematic and approximately 20% requested clarification. Participants believed they were being asked about current physical symptoms, such as “feeling bloated” or “back pain” related to colon cancer. Due to repeated types of questions for the *perceived benefits* and *perceived barriers* scales for FOBT and colonoscopy, some participants at Site A needed reminders of the procedure in question. As well, Site A participants who had screening did not see the need for screening intention items, and they also felt there were too many response categories in sub-scales. The “I neither agree or disagree” was perceived to be a good option for most participants (Site A & B).

The average time to administer the survey ranged from 15 to 30 min irrespective of whether CAPI was used or paper-pencil administration (later in administration). Survey length and interview time was perceived differently by RAs and participants. For Site A, the average time to survey completion was 20 min; although, 20% took a bit longer because they did not recall information. At Site B, 52% took 15 min during survey administration; however, perceived time was 20–30 min.

#### Evaluation of CAPI software

RAs felt it was a feasible option for interviewer-led administration; yet, issues with CAPI were identified after the first 50 surveys. The strengths included the ability to log real time responses without missing an item. The limitations were mainly associated with the computer/tablet. Purchasing a lower quality product posed several problems: (1) glare on the screen during administration at social events or outdoors; (2) small font size could not be adjusted at the software or computer/tablet level; (3) inconvenience with handling the computer/tablet while standing and maintaining privacy; and (4) errors with data entry due to combined effects of glare and font size when later cross checked. This was the rationale for paper-pencil administration alongside computer/table entry to detect potential errors but also to avoid missing data. RAs were able to find better spaces and places to administer the survey using the tablet but also collected data using the paper-pencil method. RAs determined the process of collecting data using CAPI and paper surveys; that is, to do the paper-pencil survey first and then enter it into tablet or vice versa.

#### Evaluation of resources

RAs and participants valued visual aids provided during administration; this included the FOBT kit and anatomical image of colonoscopy. When participants asked more specific CRC or screening questions, RAs responded after survey administration. Site A RA reinforced the importance of accessibility with conducting surveys in Urdu enabling them to speak in their own language with an RA from the same background. While the honorarium was perceived to be feasible and attractive, both RAs felt investing more funds towards a better tablet would improve administration and result in less error.

### Psychometric testing

#### Principal component analysis

**Fig. 2:**
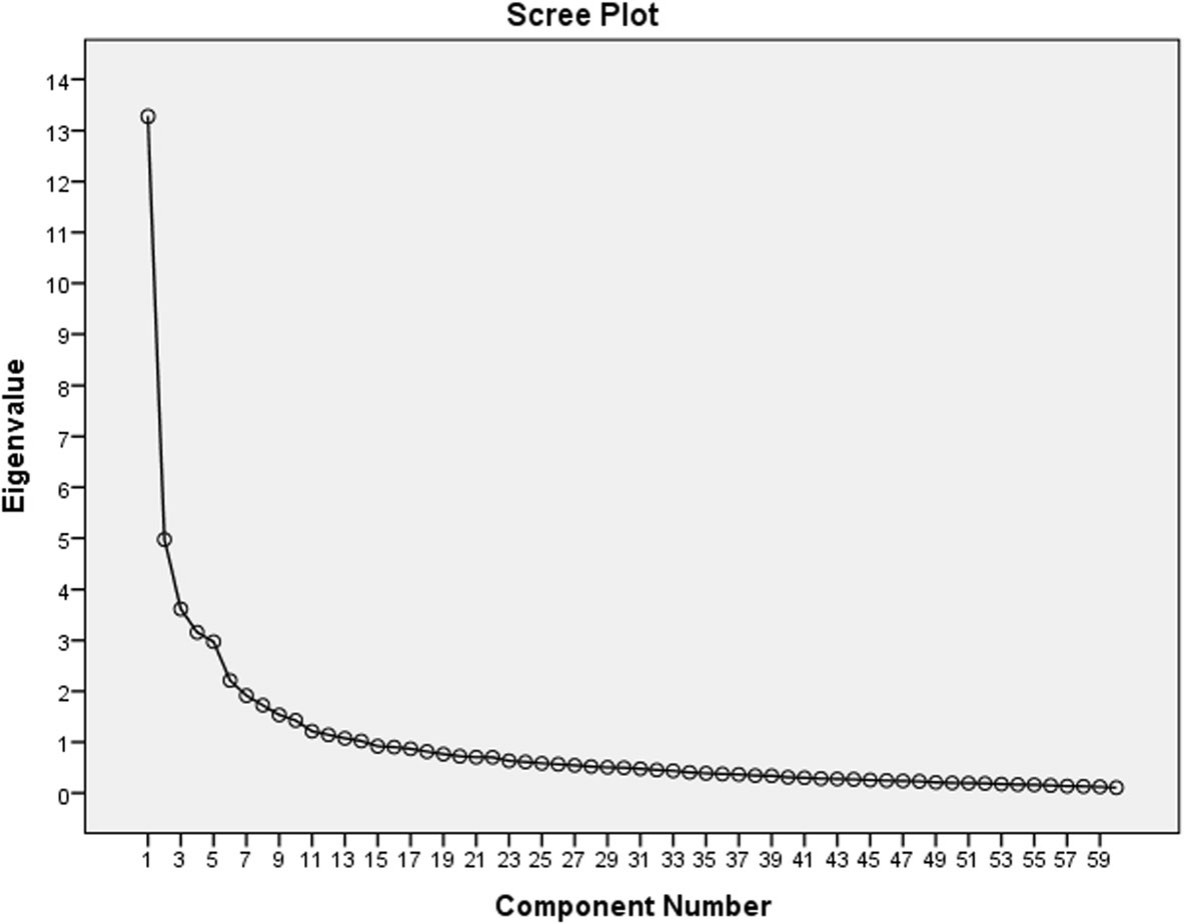
Scree plot showing elbow at number of components with Eigen values > 1. (elbow at 14 components)

Initial principal component analysis identified 14 components, and collectively, they explained 68.7% of the total variance (See Table 5). The accompanying scree plot highlights the elbow at 14 components (Fig. 2). Factor loadings on principal components that were 0.30 or higher were retained. ITC of 0.2 or less were removed, and 34 items remained, categorised into 4 sub-scales in the final scales (See Table 6).

**Table 5:** Principle components and total variance

Component	Initial Eigen values		
	Total	% of Variance	Cumulative %
1	13.275	22.125	22.125
2	4.973	8.288	30.413
3	3.617	6.029	36.442
4	3.159	5.264	41.706
5	2.974	4.956	46.662
6	2.213	3.689	50.351
7	1.915	3.191	53.542
8	1.725	2.874	56.416
9	1.534	2.557	58.973
10	1.427	2.378	61.351
11	1.213	2.022	63.372
12	1.143	1.905	65.277
13	1.074	1.789	67.066
14	1.021	1.701	68.767

**Table 6:** Retained items in 4 subscales

SUBSCALE	ITEMS
*Perceived susceptibility* [4 items]	It is extremely likely I will get colon cancer in the future.
	I feel I will get colon cancer in the future.
	There is a good possibility I will get colon cancer in the next 10 years.
	My chances of getting colon cancer are great.
*Perceived severity* [9 items]	The thought of colon cancer scares me.
	When I think about colon cancer, my heart beats faster.
	I am afraid to think about colon cancer.
	Problems I would experience if I had colon cancer would last a long time.
	Colon cancer would threaten a relationship with my partner.
	If I had colon cancer, my whole life would change.
	If I developed colon cancer, I would not live longer than 5 years.
	If I am fated to get colon cancer, I will get colon cancer; there is nothing I can do to change fate.
	Colon cancer is like a death sentence; if I get it, I will surely die from it.
*Perceived barriers* [19]	FOBT You are afraid to have a home stool test because you might find out something is wrong.
	A home stool test is embarrassing.
	You do not have time to do a home stool test.
	The cost would keep you from having a home stool test.
	You do not need to do a home stool test because you have no problems.
	You do not know how to do a home stool test.
	You do not have privacy to do a home stool test.
	Collecting a stool sample to do a home stool test is unpleasant to you.
	The doctor never told you to have a home stool test.
	Colonoscopy You are afraid to have a colonoscopy because you might find out something is wrong.
	A colonoscopy is embarrassing.
	You do not have time to do a colonoscopy.
	The cost would keep you from having a colonoscopy.
	You do not need to do a colonoscopy because you have no problems.
	You feel anxious about having a colonoscopy because you don’t really understand what will be done.
	Having a colonoscopy is painful.
	Having to follow a special diet and taking a laxative would keep you from having a colonoscopy.
	You are afraid to have colonoscopy because of the possibility there may be bleeding or tearing of the colon
	Transportation problems would keep you from having a colonoscopy.
*Subjective norm* [2 items]	I want to do what members of my immediate family think I should do about colon cancer screening.
	Members of my immediate family think I should have colon cancer screening.

#### Reliability

Internal consistency reliability analysis was completed using all retained items in 4 remaining sub-scales. See Table 7. The overall scale reliability estimate of Cronbach’s alpha for the 34 items was 0.88, 95% CI (0.86–0.90).

**Table 7:** Cronbach alpha of scales

Subscale	Cronbach alpha Statistic (95% CI)
Perceived susceptibility (4 items)	0.87 (0.84, 0.89)
Perceived severity (9 items)	0.80 (0.76, 0.83)
Perceived barriers (19 items)	0.91 (0.89, 0.92)
Subjective norm (2 items)	0.79 (0.73. 0.83)
Overall scale (34 items)	0.88 (0.86, 0.90)

#### Construct validity

After adjustment for key variables, multiple linear regression demonstrated a significant difference in the *perceived barriers* scale scores (− 12.21; 95% CI, − 17.13 to - 7.28; *p* <  0.0001) between participants who participated in screening (FOBT or colonoscopy) compared to those who did not (Tables 8 and 9). The association between years of residence between those who resided in Canada ≥10 years and ≤ 10 years, and uptake of CRC screening was not significant (OR 0.91 (0.46–1.79), *p* = 0.783) after adjustment for key variables.

**Table 8:** Construct Validity*

Association between Perceived barriers score, and screening or not screening with the “home” test (FOBT) or colonoscopy		
Dependent variable	Estimated coefficient (95% CI)	*P*-value
Perceived barriers score	−12.21 (−17.13, −7.28)	< 0.001

*Adjusted for age, years lived in Canada, gender, country of birth, ethnic background, marital status, level of education, language, employment status, individuals making up family, household income, having a family doctor

**Table 9:** Construct Validity*

Association between years lived in Canada and the uptake of CRC screening.		
Independent variable	Odds ratio (95% CI)	*P*-value
Living in Canada ≥ 10 years	0.91 (0.46 – 1.79)	0.783

*Adjusted for age, years lived in Canada, gender, country of birth, ethnic background, marital status, level of education, language, employment status, individuals making up family, household income, having a family doctor

## Discussion

The CCSBS was developed using rigorous methods [24–27] and this study evaluated the included scales for future use in survey research on CRC screening with SA populations. The results of pilot testing demonstrated good feasibility for recruitment, interviewer-led administration, and data collection with improved tablet functionality. The final survey has resulted in a shorter survey after psychometric testing. The CCSBS may be used to describe or predict CRC screening that influences uptake among SAs, and in turn, this may be useful to inform intervention planning, such as tailored approaches for SAs.

The sample in our study closely represented the demographics of SAs in Canada where approximately two thirds originated from India [40]. A greater percentage lived in Canada for > 10 years, while 21.6% lived in Canada < 10 years. We aimed to recruit more newcomers to our study; however, this was a challenge given that they were more likely to refuse to participate. CRC screening using either the home stool test or colonoscopy was high in our study reflecting potential trends among SAs; granted, these rates are not unusual given that they were self-report. Although, higher CRC screening rates among SAs have been reported by others as well. In one US study [41], Asian Indians had higher CRC screening rates within an organized program than non-Hispanic White participants. As well, a Canadian study [42] indicated that foreign-born patients in primary care practices had greater likelihood of CRC screening adherence then Canadian born peers.

The concerns raised by Site A participants regarding number of response categories was important because in prior work, a 5-point Likert scale was used [26, 27]. The decision to change to a 7-point Likert scale occurred because we wanted to have greater discriminative power [43]. What is not known is whether the response burden of the 84-item survey may have indirectly influenced Site A participant’s perceptions that the 7-point Likert response categories were a greater burden. This may have been the case as two thirds of the total items were comprised of the six sub-scales. In a simulation study, Likert scales of 4 to 6-point response categories showed similar psychometric properties [44]. However, this may not be transferable to diverse populations or context of survey administration. In smaller scales, 5- or 6-point Likert scales is required to counteract the underlying effects of scale length. In our final survey, because two scales had 2 and 4 items, we decided to maintain the 7-point Likert response categories to enhance item discrimination.

Given that 56% of participants “did not know or prefer not to answer” the item asking about household income, it raises a question of how to address this in the survey, particularly given that some SA participants may have significantly lower incomes or be living in poverty [45]. Using categories that range from < 19,000 to 40,000 or greater may be more conducive to this population. We believe that disclosure of income also may be related to similar concerns with not wanting to disclose personal information. Socio-culturally, SAs may be reluctant to share personal information with those they do not know well or trust because they may fear it being shared with other community members, or outside the research team [46]. Being familiar with the participant enabled trust to share information. Fear of sharing information with the family physician may reflect aspects of the physician-patient relationship and/or lack of trust [47]. Although, physician trust was not explored in our survey, it may be an important concept to expand upon in future study.

The use of CAPI was a positive aspect but it also had its limitations. The issue with functionality of the tablet necessitates upgrading to a better one to improve data collection. Screen visibility or computer/tablet malfunction has been cited as a limitation of the application in other studies [48]. This links also to the need to assess preference for paper-based interview-led or CAPI survey administration. Length of time to complete interview-led survey administration has also been reported as a barrier that impacts participation among SAs [46]. In our study, length of time for survey administration was not as high as participants perceived it to be. Given that the length was reduced in the final survey, this may not be an issue moving forward.

### Final scale

This pilot study assessed psychometric properties of the developed scales to verify findings from prior studies [24–27]. The final survey was reduced to 4 scales and 34 items: *perceived susceptibility*, *perceived severity*, *perceived barriers* (home stool test and colonoscopy), and *subjective norm*. There was good internal consistency of scales with Cronbach’s alpha ranging from 0.80–0.91. Construct validity confirmed that *perceived barriers* resulted in low CRC screening adherence. The second hypothesis failed to substantiate what is in the literature regarding newcomer SAs [20, 21], as long-term residence and recent residents had similar screening rates in this study. This is important to public health planning as interventions need to target all SAs not only newcomers. As well, gaining insights to facilitating and inhibiting factors that influence CRC screening enables public health and cancer care to plan strategies that ameliorate access to uptake among SAs [49].

### Strengths

The strengths of this study include the comprehensiveness of survey development [24–27] prior to pilot testing the CCSBS. The scales in the survey were reduced to 34 items, making it more manageable and reducing response burden. Survey administration mode was also a positive aspect as it contributed to ensuring completeness of data collection, overcame issues of understanding written content, and enabled access because the Urdu language in the spoken form is understandable across diverse language groups. The sample size permitted stable estimates on psychometric tests evaluated. The sample was also balanced by gender and recruitment site. As well, diversity of participants was noted in relation to highest level of education completed, income, and employment.

### Limitations

As we were unable to recruit more newcomer SAs, we acknowledge that participants in our study may have been more motivated, more likely to participate if they had CRC screening, and systematically different than newcomers or those who did not participate. The self-report rates of screening are also something to consider. Over reporting of screening, especially, using the home stool test or FOBT may have occurred [50]. We did not perform test-retest reliability, and this may be perceived to be a limitation.

## Conclusion

Community-based recruitment was feasible, as was interview-led administration of the CCSBS using CAPI if functionality of the tablet improved. Both the English and Urdu version of the surveys were tested and finalized. The survey can be used to assess CRC screening rates, intention, and factors that influence screening behaviour in SAs in similar westernized contexts. The survey may also be useful in planning tailored interventions to promote CRC screening and uptake in this diverse population.

## Data Availability

Most of data generated and analysed during this study are included in this published article. Additional files for factor analysis completed during the current study are available from the corresponding author on reasonable request.
